# A Machine Learning Classification Model for Gastrointestinal Health in Cancer Survivors: Roles of Telomere Length and Social Determinants of Health

**DOI:** 10.3390/ijerph21121694

**Published:** 2024-12-19

**Authors:** Claire J. Han, Xia Ning, Christin E. Burd, Fode Tounkara, Matthew F. Kalady, Anne M. Noonan, Diane Von Ah

**Affiliations:** 1 Center for Healthy Aging, Self-Management and Complex Care, College of Nursing, The Ohio State University, Columbus, OH 43210, USA; vonah.1@osu.edu; 2 The James: Cancer Treatment and Research Center, The Ohio State University, Columbus, OH 43210, USA; fode.tounkara@osumc.edu; 3 Clinical Informatics and Implementation Science, Biomedical Informatics (BMI), College of Medicine, The Ohio State University, Columbus, OH 43210, USA; ning.104@osu.edu; 4 Computer Science and Engineering (CSE), College of Engineering, The Ohio State University, Columbus, OH 43210, USA; 5 Departments of Molecular Genetics, Cancer Biology, and Genetics, The Ohio State University, Columbus, OH 43210, USA; christin.burd@osumc.edu; 6 Department of Biomedical Informatics, College of Medicine, The Ohio State University, Columbus, OH 43210, USA; 7 Division of Colon and Rectal Surgery, Clinical Cancer Genetics Program, The James: Cancer Treatment and Research Center, The Ohio State University, Columbus, OH 43210, USA; matthew.kalady@osumc.edu; 8 GI Medical Oncology Section, The James: Cancer Treatment and Research Center, The Ohio State University, Columbus, OH 43210, USA; anne.noonan@osumc.edu

**Keywords:** cancer survivors, gastrointestinal health, telomere, social determinants of health, machine learning

## Abstract

Background: Gastrointestinal (GI) distress is prevalent and often persistent among cancer survivors, impacting their quality of life, nutrition, daily function, and mortality. GI health screening is crucial for preventing and managing this distress. However, accurate classification methods for GI health remain unexplored. We aimed to develop machine learning (ML) models to classify GI health status (better vs. worse) by incorporating biological aging and social determinants of health (SDOH) indicators in cancer survivors. Methods: We included 645 adult cancer survivors from the 1999–2002 NHANES survey. Using training and test datasets, we employed six ML models to classify GI health conditions (better vs. worse). These models incorporated leukocyte telomere length (TL), SDOH, and demographic/clinical data. Results: Among the ML models, the random forest (RF) performed the best, achieving a high area under the curve (AUC = 0.98) in the training dataset. The gradient boosting machine (GBM) demonstrated excellent classification performance with a high AUC (0.80) in the test dataset. TL, several socio-economic factors, cancer risk behaviors (including lifestyle choices), and inflammatory markers were associated with GI health. The most significant input features for better GI health in our ML models were longer TL and an annual household income above the poverty level, followed by routine physical activity, low white blood cell counts, and food security. Conclusions: Our findings provide valuable insights into classifying and identifying risk factors related to GI health, including biological aging and SDOH indicators. To enhance model predictability, further longitudinal studies and external clinical validations are necessary.

## 1. Introduction

Cancer is the second leading cause of death in the United States, following heart disease. Projections for 2024 estimate 2.0 million new cases and 611,720 cancer deaths [1]. Encouragingly, medical advancements have increased survival rates among cancer patients [2,3]. However, cancer survivors often face multiple short- and long-term side effects throughout their treatment [2,3]. These effects include physical (e.g., pain, neuropathy, functional limitations), gastrointestinal (GI), and mental health (e.g., depression, anxiety) concerns [2,3,4]. The prevalence and severity of these physical, GI, and mental health issues can vary widely, impacting survivors’ health-related quality of life (HRQOL), treatment adherence, daily functioning, nutrition, and overall prognosis. Addressing these health concerns is essential for enhancing the long-term well-being of cancer survivors.

Notably, GI symptoms often persist in cancer survivors even after treatment completion. These symptoms include nausea/vomiting, appetite loss, altered bowel movements (e.g., diarrhea or constipation), bloating, indigestion, heartburn, and abdominal pain [5,6,7,8,9]. GI symptoms rank as the most common chronic physical side effects of cancer treatments, following psychological distress and fatigue in cancer survivors with mixed cancer types [10]. In a study of 142 breast cancer survivors, the GI symptom cluster was the second most prevalent after chemotherapy [11]. In a cohort of 413 colorectal cancer survivors, 81% experienced persistent GI symptoms eight years post-treatment [8]. A review of GI toxicity after radiotherapy in rectal cancer survivors indicated that long-term GI toxicity continued for over three months, with symptoms including diarrhea (35%), fecal incontinence (22%), abdominal gas (71%), and abdominal pain (13%) [12].

GI side effects related to cancer treatments are particularly prevalent among older adult cancer patients, impacting physical and social functioning as well as HRQOL [13]. The GI symptoms are significant concerns for older adult cancer patients, with the incidence of overall GI symptoms having been reported to be as high as 40% in cancer patients receiving standard-dose chemotherapy and 100% in those on high-dose chemotherapy [14]. Several factors contribute to the increased prevalence of GI issues in this population. Firstly, the aging process significantly affects oropharyngeal motility, upper esophageal motility, colonic function, and GI immunity [15]. Secondly, older adults often have comorbidities and long-term exposure to medications, alcohol, and tobacco that may exacerbate GI distress [16]. Furthermore, cancer treatments can induce accelerated aging in individuals with cancer [17]. Mechanisms such as oxidative stress, inflammation, and mitochondrial dysfunction are implicated [17]. This accelerated aging phenomenon can worsen existing GI health conditions. Consequently, GI health concerns may be associated with the aging process, making cancer survivors more vulnerable to these connections.

Recent studies underscore the increased significance of biological over chronological aging in the physical and psychological well-being of cancer survivors [18,19,20]. Notably, telomere length (TL), which shortens during cell division, is a validated measure of biological aging [21,22]. In individuals of the same chronological age, shorter TL is linked to accelerated biological aging and various health conditions in cancer survivors [20,21]. While the association of TL with survival and mortality is well studied in cancer survivors [22], its relationship with HRQOL, including GI health, requires further investigation [18,19]. Social determinants of health (SDOH) significantly impact the physical and mental health of cancer survivors. Factors such as race/ethnicity, socioeconomic status, education, and marital status play crucial roles in the health outcomes of cancer survivors [23,24]. Chronic stress associated with poor SDOH triggers systemic inflammation, exacerbating physical symptoms [25,26]. Moreover, there is a potential link between TL, SDOH, and inflammation [27]. Poor SDOH status has been associated with TL shortening due to chronic stress and inflammation in US adults living in the community [27]. Therefore, SDOH and TL may be related to GI health in this population. Understanding this complex interplay could inform interventions to improve GI health in cancer survivors.

The classification of GI health conditions and identification of contributing factors are crucial steps in selecting and applying personalized interventions for cancer survivors [28,29]. Machine learning (ML) offers substantial advantages in cancer survivorship care, particularly in classification or prediction models [30,31]. Unlike traditional statistical methods, ML can handle small sample sizes and multiple variables with complex relationships by controlling covariates and multicollinearity. It excels at identifying intricate patterns, managing high-dimensional data, and adapting over time [30,31]. This capability is especially beneficial in cancer survivorship research, where the number of survivors for certain cancer types may be limited and the relationships among cancer treatments and health outcomes can be complex [28,29]. While ML has been employed to develop predictive models for cancer diagnosis and survival [32], its application to GI health conditions in cancer survivors remains relatively rare.

Therefore, by leveraging ML with high precision, we aimed to develop and validate an ML classification model of GI health conditions (better vs. worse), incorporating TL and SDOH indicators as our primary interests, along with demographic and clinical characteristics, including inflammatory markers, as secondary interests. The current study is a pilot to explore and identify significant features, including biological aging markers (i.e., TL in our study) and SDOH indicators, to classify GI health conditions in adult cancer survivors, not limited to those over 65. This approach enhances the performance of ML classification models by increasing sample size and providing a comprehensive understanding of GI health across different age groups.

## 2. Methods

### 2.1. Sample and Procedures

**Figure 1:**
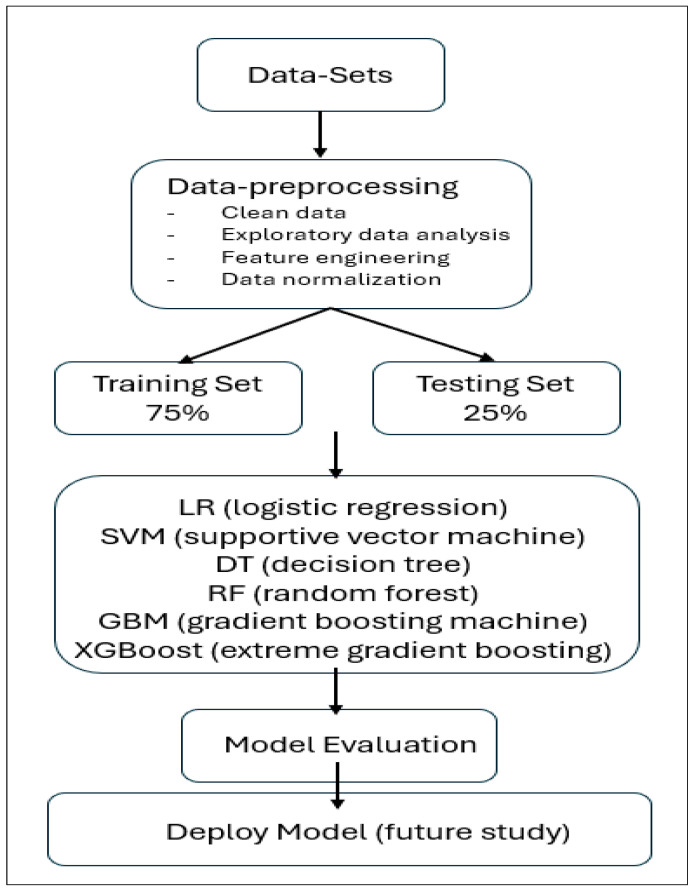
Data processing. This figure illustrates the comprehensive process of machine learning data processing. Model deployment is suggested in a future study.

In this secondary analysis, we utilized data from the National Health and Nutrition Examination Survey (NHANES), conducted by the National Center for Health Statistics (NCHS) under the Centers for Disease Control (CDC) [33]. The NHANES dataset includes cross-sectional, nationally representative health and nutritional data from 21,004 non-institutionalized US civilians aged 2 months and older [33]. We chose to use the NHANES dataset from 1999–2002 because it includes TL data from leukocyte DNA samples, available for those years. At the time of our study, this was the most recent NHANES dataset available with TL data for participants aged 20 years of age and above. Of the 10,291 participants aged 20 or older, 7827 (76%) had TL data. Among the participants with TL data, 645 with self-reported cancer diagnoses were included in the current study. Given the strong relationships between chronic non-cancer GI disorders (e.g., functional GI disorders and liver diseases), we excluded eligible samples with chronic non-cancer GI disorders. We also applied sampling weights in the analysis to address oversampling and non-response biases, ensuring the accuracy of estimates reflecting the broader US population [34]. We employed a standard mining approach consisting of four stages: (1) data acquisition, (2) preprocessing (e.g., data cleaning, exploratory data analysis addressing class imbalances, optimizing dataset classes with feature engineering, and data normalization), (3) model learning with training and testing data, and (4) model evaluation [35] (Figure 1).

### 2.2. Features

#### 2.2.1. Demographic and Clinical Data, Including Inflammatory Markers

Information on chronological age (years), sex (male, female), and comorbidities including hypertension, diabetes, cardiovascular disease, and history and types of cancer diagnosis were collected from self-reported questionnaires. White blood cell count (WBC) was extracted from complete blood count (CBC) data retrieved from the “Complete Blood Count with 5-part Differential—Whole Blood” category of the 1999–2002 NHANES laboratory data [36]. C-reactive protein (mg/dL) levels were measured by high-sensitivity, latex-enhanced nephelometry by the Immunology Division, Department of Laboratory Medicine, University of Washington Medical Center. As diet is relevant to GI health, we also included diet data [36].

#### 2.2.2. Telomere Length (TL) Measurement

The measurement of TL in the NHANES study has been published elsewhere [37,38,39,40]. Detailed protocols describing TL measurement for the NHANES study are accessible on the CDC website under the laboratory section [41]. In brief, TL was assessed by isolating purified DNA from whole blood samples using the Puregene (D-50 K) protocol (Germantown, Maryland). The TL assay was conducted via polymerase chain reaction. TL was measured relative to standard reference DNA (T/S ratio), with each sample being analyzed three times on different days and in duplicate wells, totaling six data points. Potential outliers (<2% of samples) were identified and excluded. The inter-assay coefficient of variation was 6.5% [27]. The provided values represent the mean (standard deviation) of the T/S ratio. The CDC Institutional Review Board granted human subject approval for TL measurements, which were quality-controlled before being linked with the NHANES database.

#### 2.2.3. Social Determinants of Health (SDOH)

We included nine selected SDOH variables based on sociodemographic variables and cancer health risk behaviors identified in the CDC Healthy People 2030 SDOH framework [42] and in the National Academy of Medicine [43,44,45]. The nine risk factors corresponding to SDOH domains included in our study were: (i) racial/ethnic minorities; (ii) low educational achievement (i.e., less than a high school diploma or equivalent); (iii) poverty–income ratio (i.e., family income/poverty threshold; a ratio <1 indicates that annual family income is below the poverty level); (iv) food insecurity as per the Kendall/Cornell scale (i.e., low or very low food security) [46]; (v) current smokers (individuals who have smoked a minimum of 100 cigarettes in their lifetime and who currently smoke either daily or occasionally); (vi) heavy drinkers (as per the Alcohol Use Disorders Identification Test-Concise [AUDIT-C] screening tool, with >4 for males and >3 for females indicating a moderate risk of alcohol abuse) [47]; (vii) low physical activity (engaging in less than 10 min of moderate or intense activity or strength training in the past month); (viii) lack of a partner (divorced/widowed/single marital status) [45]; and (ix) diet quality. In the NHANES 1999–2002 datasets, dietary information was collected using 24 h recalls [37]. Using these NHANES data, we included major food groups—total energy in kcal, total protein, total carbohydrate, total saturated fat, total fatty acid, total sodium, and total fruit and vegetables—based on the US Dietary Guidelines for Americans in our study [38]. We further computed overall diet quality using the Healthy Eating Index (HEI) 2015 [37,39]. The overall HEI-2015 score varies from 0, indicating nonadherence, to 100, indicating perfect adherence. There is no established minimal clinically significant difference for the HEI-2015 [37,39].

### 2.3. Outcome

#### GI Health

We evaluated GI health status (i.e., worse or better status) using the Health Status Questionnaire, which includes the CDC health-related quality of life (HRQOL)-4. The HRQOL-4 shows high reliability (0.57–0.75) in the general population and among cancer survivors [48]. For GI health conditions, participants were asked to answer: “Did you have a stomach or intestinal illness over the last month?” (no—better GI health versus yes—worse GI health).

### 2.4. Data Analyses

#### 2.4.1. Initial Data Analysis (For Feature Selection)

We included potential variables related to GI health among cancer patients based on the literature [7,8] and statistical analyses. For descriptive analyses, categorical variables were presented as counts and percentages, while continuous variables were presented as means and standard deviations. We primarily compared the training and test datasets using two-sample independent *t*-tests or chi-square tests. Additionally, we examined the initial associations of input data with GI health (better versus worse) using two-sample independent *t*-tests or chi-square tests.

#### 2.4.2. Machine Learning Model

*Data preprocessing:* We only included input variables showing significant associations with GI health in our initial data analyses [31,49]. In feature engineering, we identified and selected the most relevant features of GI health based on the literature [7,8]. Among the 645 adult cancer survivors used to build the model, all had TL data, and most of the employed features were available with low missing data rates (missing data rates < 4%). Because the rates of missing data in our study were trivial, we applied complete case analyses. We identified and removed outliers using the interquartile range (IQR) and eliminated duplicate entries to avoid skewing the models. Next, we performed data transformation, including normalization (scaling numerical features to a standard range of 0–1) to ensure all features contribute equally to the models and standardization to a mean of 0 and a standard deviation of 1, reducing feature dimensions and enhancing the performance and stability of our ML models [49]. We employed ordinal encoding for categories with a natural order.

*Classification modeling:* We first created a training dataset by randomly matching 75% of all cancer survivors in the dataset and created a test dataset with the remaining 25% of cancer survivors (Figure 1). Python’s train_test_split function from the scikit-learn (sklearn) library was employed to randomly assign samples to either the training or testing set based on the average prevalences of better GI health, ensuring dataset integrity [30]. The training dataset had 484 cancer survivors, and the test dataset had 161 cancer survivors. We utilized the training dataset for initial modeling and the test dataset to assess the model’s performance in classifying GI health on unseen data. Considering the number of features in the 645 sample size, we applied six supervised ML modeling methods, including logistic regression (LR), support vector machine (SVM), decision tree (DT), random forest (RF), gradient boosting machine (GBM), and extreme gradient boosting (XGBoost) [30]. Each method was assessed with a specific learning algorithm to determine its effectiveness in classifying GI health conditions. Logistic regression, a widely used binary classifier, served as the baseline model for comparison. Hyperparameter tuning was conducted through random search with 5-fold cross-validation to prevent overfitting. The 5-fold cross-validation was evaluated using the mean value of each model performance index with a 95% confidence interval (CI) [49]. Hyperparameters such as tree complexity, learning rate, and number of trees were adjusted for RF, GBM, and XGBoost. Linear kernel functions were favored over nonlinear ones, like the radial basis function kernel, in support vector machine models to avoid overfitting in a small dataset (Supplementary Materials) [49]. The prediction model performance was evaluated using various metrics, including accuracy, precision, recall (sensitivity), specificity, F1 score, and area under the receiver operating characteristic curve (AUC). Once the final model performance metrics were achieved, we employed McNemar’s statistical test to compare the performance of the proposed models. As our study focuses on identifying the optimal AI model selections, we only compared the top two models with the highest values in each performance index.

*Permutation feature importance (PFI)*: Feature importance analysis identified and ranked the most influential features for classifying GI health conditions [28]. We used PFI to quantify the impact of each feature by calculating their contribution ranks in our final best-performing machine learning models. This was based on model performance indices. By employing the trained classification model, each feature was randomly shuffled 10 times individually, and the resulting decrease in model accuracy was measured. The importance was determined using the hold-out test data to highlight the impact of features on GI health. We controlled for other input features that showed significant relationships with GI health in our ML models.

A significance level of *p* < 0.05 was applied, and the overall statistical analyses were carried out using R software (version 3.6.3, R Foundation for Statistical Computing, Vienna, Austria). Furthermore, the training and test sets were split using the sklearn.model_selection.train_test_split function. The RF data were analyzed using the sklearn.ensemble.RandomForestClassifier module. The SVM data were analyzed using the sklearn.svm.SVC module. Cross-validation was performed with the sklearn.model_selection.cross_val_score function. PFI was conducted with the scikit-learn function. All of these packages were utilized within Python (version 3.10.2, Python Software Foundation, Wilmington, NC, USA).

### 2.5. Conceptual Framework

**Figure 2:**
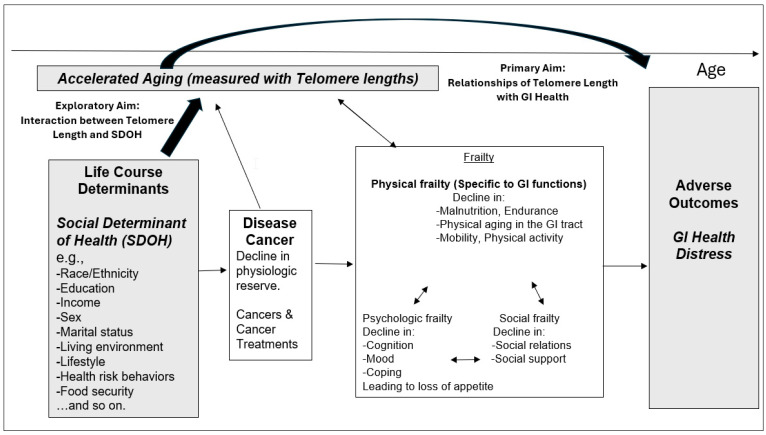
Integral conceptual model of frailty, adapted to the current study. We have obtained permission to revise the original framework and adapt it to the current study, ensuring compliance with copyright regulations. Both reproduction and adaptation of the original framework have been granted copyright permission (Supplementary Materials).

The conceptual framework (Figure 2) for this study is based on the original framework, the integral conceptual model of frailty [50]. Figure 2 displays this framework, which includes various factors (e.g., life course determinants) and diseases (e.g., cancer and cancer treatment) that may impact frailty, which is correlated with accelerated aging. This impact is seen in the model’s sub-dimensions, namely physical, psychological, and social frailty. These three sub-dimensions can be characterized by a decline in various factors. Specifically, a decline in nutrition, mobility, physical activity, and physical function in the GI tract is relevant to GI health. Increases in frailty (specifically physical GI function in our study) ultimately result in adverse events (i.e., GI distress in our study). We used TL as a proxy for accelerated aging, which is impacted by SDOH as well as cancer disease and its treatment. Our review analyzed and synthesized the data by mapping TL (accelerated aging), SDOH (life course determinants), and GI health (adverse events) in adult cancer survivors to elements of the integral conceptual model of frailty [50]. In our study, we focused on the components represented by the grey boxes in this framework (Figure 2).

## 3. Results

### 3.1. Initial Descriptive Analyses

#### 3.1.1. Participant Characteristics, Clinical Data, SDOH, and GI Health

Table 1 describes the participant demographic and clinical characteristics, SDOH, and GI health conditions of both the training (N = 484, 75% of the total sample N = 645 adult cancer survivors) and the test (N = 161, 25% of the total sample N = 645 adult cancer survivors) datasets. The mean participant age was 66.3 ± 14.7 years for the training dataset and 65.5 ± 16.2 for the test dataset (*p* = 0.102). Approximately half of the participants were women, and skin cancer (approximately half were melanoma) was the most common cancer type in both datasets. WBC, CRP, and TL did not significantly differ between the training and test datasets. A total of 67.5% of individuals reported better GI health in the training dataset, while 62.7% reported better GI health in the test dataset (*p* = 0.412). Our data demonstrated a normal distribution among continuous variables in each training and test dataset (Supplementary Materials). Most participants were non-Hispanic Whites, married, and had an education level of high school or below. Cancer risk behaviors (such as smoking, heavy alcohol consumption, physical activity, and diet quality score—HEI) were not significantly different between the two datasets. The prevalences of participants’ annual family incomes ranging below the poverty level and experiencing food insecurity were similar in both datasets (39.7% versus 37.3%, *p* = 0.423; and 8.6% versus 8.1%, *p* = 0.879, in the training dataset versus the test dataset, respectively).

#### 3.1.2. Potential Risk Factors for GI Health Within the Training Dataset

Before developing the ML model for GI health, we examined the associations between potential risk factors, including demographics, clinical data (including inflammatory markers, TL, and SDOH), and GI Health in the training dataset. Table 2 summarizes the results of these comparisons between cancer survivors with better and worse GI health. The better GI health group was younger (63.3 years vs. 66.4 years, *p* = 0.031), contained a smaller proportion of females (47% vs. 65%, *p* = 0.013), and had fewer comorbidities (41% vs. 45%, *p* = 0.043). Mean WBC (k/ul) and CRP (mg/dl) levels were also lower in the better GI health group. The mean TL (kb) was longer in the better GI health group. Non-Hispanic Whites were more prevalent in the better GI health group (80.3% vs. 77.3%). Hispanic individuals were more prevalent in the worse GI health group (10.7% vs. 7.5%, *p* = 0.039). Higher income levels were associated with better GI health (*p* = 0.038). In terms of cancer risk behaviors, heavy alcohol users (21.3% vs. 15.2%, *p* < 0.001) were more prevalent in the worse GI health group compared with the better GI health group. Cancer survivors with regular physical activity (38.5% vs. 58.3%, *p* = 0.035) and better diet quality (7.5% vs. 5.6%, *p* = 0.038) were more likely to have better GI health status compared with those with worse GI health status. The worse GI health group had higher prevalences of having income below the poverty level (37.6%) and experiencing food insecurity (8%) compared with the better GI health group. Marital status, education, BMI, and current smoking status were not associated with GI health status.

### 3.2. Machine Learning Models for GI Health

#### 3.2.1. Performance Comparison for Classification Models

**Figure 3:**
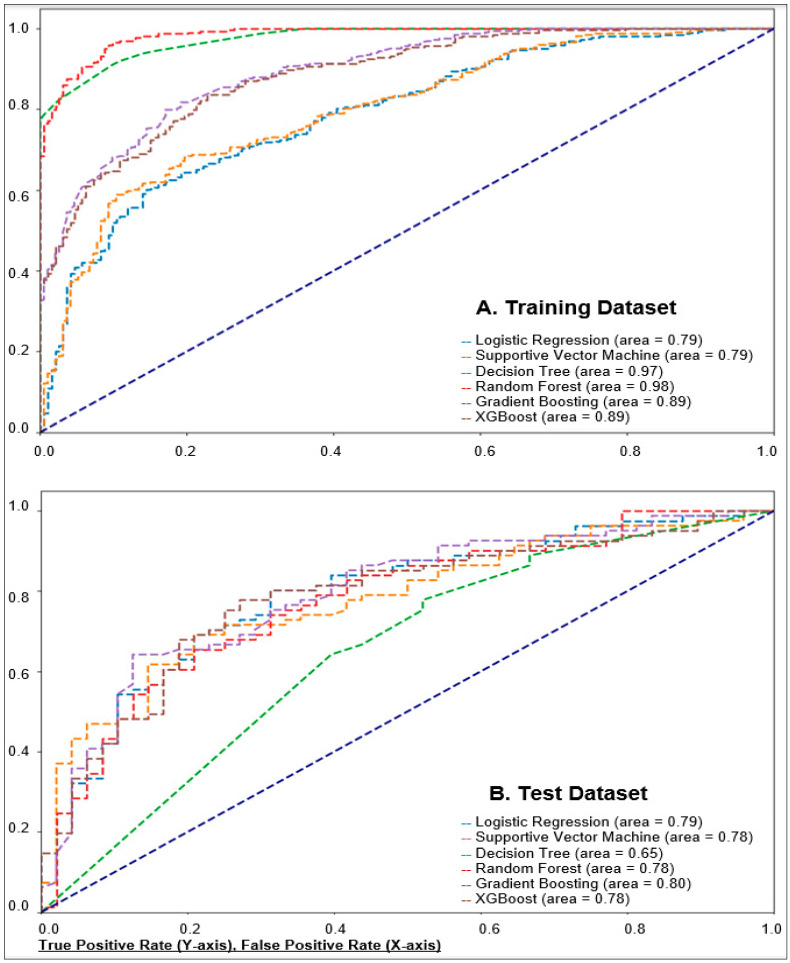
Receiver operating characteristic (ROC) curves and area under the curve (AUC) for model performance. The ROC curve is a graphical representation of a model’s diagnostic ability, plotting the true positive rate (TPR) against the false positive rate (FPR). Both graphs plot the true positive rate (TPR) on the *y*-axis, ranging from 0 to 1, and the false positive rate (FPR) on the *x*-axis, also ranging from 0 to 1. The AUC in ROC curves represents the probability that the classifier will rank a randomly chosen positive instance higher than a randomly chosen negative one. An AUC close to 1 indicates high model accuracy, 0.7 ≤ AUC < 0.8 indicates good and moderate model accuracy, 0.5 ≤ AUC < 0.7 indicates poor performance, and AUC < 0.5 indicates failure. XGBoost = extreme gradient boosting.

We present the model performance evaluation indices of all ML models using the training dataset as a development phase and the test dataset as a validation phase (Table 3). This evaluation was conducted with five-fold cross-validation. Furthermore, Figure 3 illustrates and compares the AUCs for each model in the training and test datasets. In the training dataset, random forest (RF) performed well across multiple metrics with a higher AUC (0.9842), accuracy (0.9341), sensitivity (0.9783), and F1 score (0.9489) compared with other models (Table 3). In the test dataset, the gradient boosting machine (GBM) model showed the best performance with the highest AUC (0.8035), accuracy (0.7442), precision (0.7792), specificity (0.7626), and F1 score (0.8092) across six AI model algorithms, indicating good overall performance in distinguishing between positive and negative cases. The McNemar’s statistical test used to compare the performance of the proposed models confirmed the statistical significance of the performance indices across the different AI model algorithms (Supplementary Materials).

Generally, all evaluated methods performed similarly well in DT and RF models in the training dataset and RF and GBM models in the test dataset. In the training dataset, LR and SVM models showed moderate performance with balanced metrics, but their specificity was relatively low. DT and RF models exhibited high performance across all metrics, indicating strong predictive capabilities. GBM and XGBoost models also performed well, with GBM showing good AUC and sensitivity results, while XGBoost had high precision but lower sensitivity and specificity. In the test dataset, LR and SVM maintained consistent performance from training to test datasets, with moderate accuracy and precision. DT performance dropped significantly in the test dataset, particularly in AUC and specificity. RF and GBM models continued to perform well, with RF showing strong sensitivity and GBM maintaining a good balance across metrics. Overall, the RF model in the training dataset and the GBM model in the test dataset demonstrated the most robust and reliable performance across model performance metrics.

#### 3.2.2. Feature Importance

**Figure 4:**
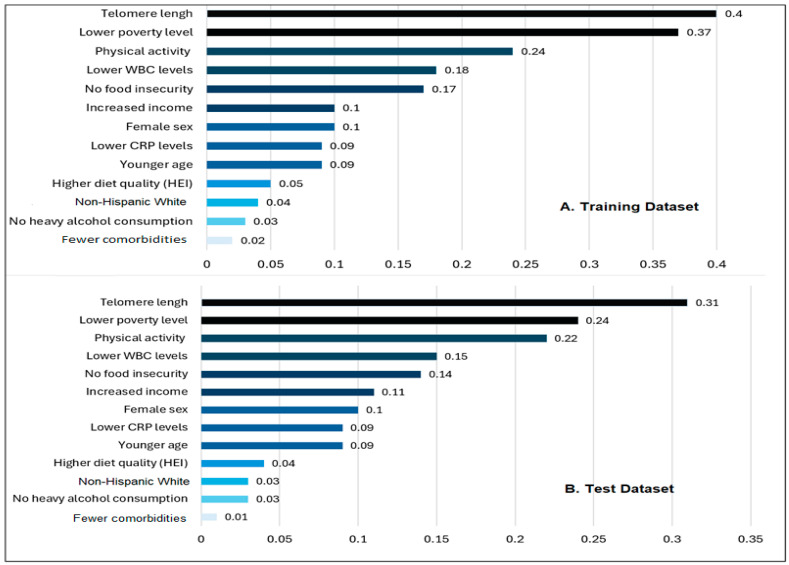
Feature importance. The bar graph indicates the positive associations of input features with a better GI health condition. The height of the bar graph of the feature importance represents the importance of the feature, with relative importance being compared with the importance values of other features to understand their significance. The feature importance scores were based on the permutation feature importance (PFI) method. Interpretation of this graph should be based on a relative comparison of the values.

The most significant features of the best-performing models in each dataset (the RF model in the training dataset and the GBM in the test dataset) were separately ranked using permutation feature importance (Figure 4). Figure 4 illustrates the relative importance of input features included in the ML models. Among the 13 input features, excluding non-significant factors of GI health in each RF and GBM model, the important top features for GI health were similar between the training and testing datasets. Incorporating TL (a value of feature importance) and several SDOH indicators in our ML models proved that their relative importance significantly contributed to classifying GI health conditions, achieving good classification performance and demonstrating potential high predictive accuracy for both the training and test datasets. Among patient demographic and clinical input variables, TL, WBC, CRP, sex, chronological age, and comorbidities contributed to GI health in both the training and test datasets. Among SDOH input variables, poverty level, food security, race/ethnicity, lifestyles, and income contributed to GI health in both the training and test datasets, with food security and poverty level being the most impactful factors on GI health. A longer TL was the most influential feature, followed by a lower poverty level. Physical activity, lower WBC, and food security were among the top ranked features of importance for better GI health in both datasets. While several other features (e.g., female sex, higher income, lower CRP, younger age, higher diet quality, non-Hispanic White group, no heavy alcohol consumption, and fewer comorbidities) play a role in better GI health, their impact is comparatively modest in our ML models.

## 4. Discussion

This study is the first to develop and validate ML classification models for GI health in adult cancer survivors, utilizing supervised ML approaches to account for multiple factors. Although we employed cross-sectional data, the ML algorithms constructed classification models based on demographics and clinical characteristics, including inflammatory markers, TL, and SDOH factors for GI health, achieving good (0.5 ≤ AUC < 0.7) to moderate to high (>0.7) prediction accuracy [29,30,31,49]. We also identified the relative importance of features in classifying GI health conditions, demonstrating that TL and certain SDOH features (e.g., economic status, and lifestyle choices) significantly influence the classification outcome (better vs. worse GI health status). The ML models developed and validated in our study could inform personalized approaches to identify cancer survivors at high risk for long-term GI distress, thereby facilitating tailored interventions that address unmet needs contributing to GI distress in adult cancer survivors.

Despite various predictive ML models being utilized in cancer survivors, such as those predicting cancer diagnosis risk, survival rates, or detecting psychological symptoms [51,52,53,54], few studies have applied ML algorithms to classify or predict GI health in this population. Previous research has identified risk factors for GI distress in cancer survivors [7,8], but these studies did not explore the associations of TL and SDOH with GI distress. Emerging evidence supports the impact of SDOH [55,56] and biological age [56,57] on symptom disparities and HRQOL in cancer survivors. Our study addresses this gap by demonstrating the feasibility of using ML approaches to classify GI health. Specifically, we investigate how TL and SDOH factors contribute to GI health in cancer survivors, providing new insights in this area. The ML models can effectively handle numerous features, minimizing both Type I and Type II errors in multiple comparisons—an advantage often not feasible in traditional statistical methods (e.g., regressions and univariate analyses). Furthermore, ML models predict or classify GI health conditions more accurately than traditional statistical methods by leveraging large datasets and complex relationships among multiple input features.

Our findings suggest that not all features contribute equally to classifying GI health conditions. TL was identified as the most influential factor in GI health, independent of chronological age, indicating a potential role for biological aging in GI conditions. The results of our study reveal positive relationships between better GI health, younger age, and longer TL. Additionally, having an income above the poverty level and engaging in routine physical activity significantly contributed to better GI health.

Telomeres, protective caps at the ends of chromosomes, play a crucial role in cellular aging [39]. Beyond chronological age, shorter TL lengths are associated with cellular senescence, where cells lose their ability to divide and function properly. Furthermore, senescent cells release inflammatory molecules, contributing to chronic inflammation associated with GI disorders such as inflammatory bowel disease, altered bowel patterns, abdominal pain, indigestion, bloating, nausea, and gastroenteritis [19,58]. Biological aging influences gut health by impairing the integrity of the intestinal barrier, affecting immune cell function, and impacting gut microbial diversity [19,58]. Our findings suggest that biological age may better reflect the functional aging of the GI tract compared with chronological age [59]. Wang et al. [19] similarly discovered that longer leukocyte TL was associated with better GI function in patients with functional GI disorders. Investigating the mechanisms responsible for the shorter leukocyte TL observed in these contexts could provide insights into managing GI health beyond considerations of chronological age in cancer survivors.

The poverty–income ratio (PIR) was the second most significant feature of GI health in our study; other SDOH variables—such as lower income levels and racial/ethnic minority status—were also associated with worse GI health. Previous studies support our findings that socially and economically vulnerable populations are exposed to more chronic stress, which can accelerate aging and promote a pro-inflammatory state in the body [60,61]. Furthermore, these vulnerable populations often face challenges in accessing healthcare resources, including community health services, oncology care, and primary care providers (PCPs). Additionally, they are more likely to reside in unsafe environments and neighborhoods, contributing to housing and food insecurity [62]. Collectively, these risk factors can lead to various forms of GI distress in cancer survivors [61].

Cancer risk behaviors, including lifestyle choices, smoking, and alcohol consumption, have well-documented associations with physical and psychological symptoms and HRQOL in cancer survivors [24,63,64]. However, limited research has explored the specific relationships between these risk behaviors and GI health in cancer survivorship. Our findings reveal that cancer risk behaviors significantly impact GI health conditions. Although previous research has primarily focused on other aspects of survivorship, such as HRQOL and psychological symptoms [24,63,64], our study highlights the need to consider GI-specific factors. The identification of risk behaviors associated with GI health provides actionable insights for survivor care. Notably, food security emerged as a more significant feature of GI health compared with self-reported diet quality as measured by the HEI. This discrepancy in feature importance for GI health could be attributed to several factors. First, self-reported diet quality may not fully capture nutrient intake or align with actual dietary behaviors [65]. Some individuals may report high diet quality despite lacking essential nutrients [65]. Second, food insecurity is prevalent among cancer survivors in the US (ranging from 4% to 83.6%) and directly influences nutrient intake, beyond broader social determinants such as poverty and health literacy [66,67]. Furthermore, food insecurity induces stress, which can exacerbate the risk of GI diagnoses, including GI cancers and disorders, by impairing gut mobility, immune responses, and barrier function [68,69]. Access to a diverse range of healthy foods ensures essential nutrients and greater microbiome diversity, which are vital for overall well-being, including GI health [68,69].

Clinical implications: ML plays a crucial role in classifying or predicting GI health, particularly for socially vulnerable cancer survivors. ML models analyze data from cancer survivors to identify those at greater risk of GI distress. Once identified, targeted interventions can address their unmet needs, whether through pharmacological or non-pharmacological approaches. Integration ML algorithms into platforms like mobile apps or websites (such as MyChart) is a practical approach. Users can access personalized insights about their GI health, receive recommendations, and make informed decisions based on ML-driven risk classifications. ML models can further tailor interventions for high-risk groups by considering their specific social needs and vulnerabilities. For example, routine assessment of accelerated aging in cancer survivors could be essential for overall well-being and GI health. Addressing smoking cessation, promoting healthy lifestyles (including a healthy diet and physical activity), and minimizing alcohol consumption could also positively impact GI health and serve as an anti-aging strategy. Lastly, routine screening for socio-economic needs may contribute to optimal GI health in cancer survivors. For instance, oncologists or PCPs can refer patients to nutritional education or food assistance programs. Increasing multidisciplinary collaboration with social workers, nutritionists, and community resources is warranted not only for overall HRQOL but also for GI health. Future investigations could further consider investigating cancer survivorship quality and survival rates based on groups classified according to our AI models.

Strengths and limitations: The strengths of our study lie in the inclusion of diverse input data, specifically inflammatory markers, TL, and SDOH features. Additionally, our focus on GI health—an unexplored area in cancer survivorship—along with the application of ML models contributes to the development of powerful classification models for GI health that consider both biological and social mechanisms. The findings of our study also reflect the importance of biological age in GI health conditions, applicable to all adult cancer survivors, not just older adults. Furthermore, our ML model was validated using an independent test dataset. However, our study has several limitations. First, NHANES is a cross-sectional survey, which may limit the predictability of our ML model. To enhance predictability, longitudinal studies with predictors and GI health conditions measured at different time points are needed. Second, the usefulness of inflammatory markers (WBC and CRP) for classifying GI health remains unclear in our study. One possible reason is that mean WBC and CRP levels fell within the clinically normal range in our samples. Third, findings regarding prediction performance should be interpreted with caution due to the overall small sample size of the test dataset. Furthermore, we acknowledge the differences in model performance metrics, such as F1 scores between the training (0.9) and test (0.8) sets in the RF models and relatively low specificity across all methods in both training and testing datasets, which suggest potential overfitting. For the differences in model performance metrics between the training and test datasets, the training data may not fully represent the variability in the test data including unseen data, leading to reduced generalization. To address this limitation, we suggest the following future directions: collecting more diverse and larger sample sizes of training data to better represent the test set, considering new features or transforming existing variables to better capture underlying patterns, and adjusting hyperparameters to reduce overfitting. For the relatively low specificity across the AI models, a higher number of positive cases may bias the model. Fourth, using a single question to assess GI health may limit our ability to fully capture GI health conditions. Furthermore, the roles of SDOH in the relationships between TL and GI health remain unknown. Lastly, cancer-related clinical characteristics, such as cancer stages, years since diagnosis, and types of treatments, were not available in our samples, although they are potential covariates for our ML models related to GI health. Future replication studies with prospective cohorts including more cancer-related clinical data are warranted to confirm the findings of our study.

## 5. Conclusions

Using an ML approach to develop and validate GI health classification models (better vs. worse) that include TL and SDOH is feasible among cancer survivors. Overall, the RF- and GBM-generated models showed the best accuracy for GI health classification. This finding suggests the potential of ML to further develop a longitudinal prediction model for GI health. TL and poverty status were the most significant features used to classify GI health and could be implemented to prevent and manage GI conditions in cancer survivors. We suggest including biological markers and SDOH in ML GI health models to optimize classification accuracy. Future longitudinal studies and external clinical validations are warranted to confirm our findings and improve model predictability.

## Figures and Tables

**Table 1:** Patient characteristics, clinical data, SDOH, and GI health.

Total Cancer Survivors (N = 645)	Training Set ^a^ (n = 75% of Total Sample, n = 484)	Test Set ^b^ (n = 25% of Total Sample, n = 161)	*p*
Age (years), mean ± SE (range)	66.3 ± 14.7 (21–85)	65.5 ± 16.2 (22–85)	0.102
Female (n,%)	235 (49.5)	84 (50.7)	0.311
Modified comorbidity index (≥2) (n,%)	168 (42.3)	66 (43.2)	0.122
Types of cancers (n,%)	Skin: 152 (21.2)	Skin: 44 (27.3)	0.143
	GU: 102 (21.0)	Breast: 35 (21.7)	
	Breast: 75 (15.6)	GU: 30 (18.6)	
	Ovary–Uterine: 45 (9.3)	Head and Neck: 21 (13.0)	
	Head and Neck: 42 (8.6)	GI: 15 (9.3)	
	GI: 41 (8.4)	Ovary–Uterine: 8 (5.0)	
	Lung: 15 (3.1)	Lung: 5 (3.1)	
	Hematological: 12 (2.5)	Hematological: 3 (1.9)	
Body mass index (BMI), kg/m^2^, mean ± SE	30.1 (0.2)	31.2 (0.3)	0.982
BMI, kg/m^2^ (n, %) < 25	170 (35.2)	56 (34.7)	0.675
25 ≤ BMI < 30	160 (33.1)	57 (35.1)	
30 ≤ BMI	153 (31.7)	48 (30.2)	
WBC (k/μL), normal (4–11 k/μL), mean ± SE	7.02 (2.1)	7.04 (2.0)	0.192
CRP (mg/dL), normal (<0.3 mg/dL), mean ± SE	0.51 (0.9)	0.62 (1.4)	0.124
Telomere lengths (kb), mean ± SE	0.93 (0.2)	0.93 (0.2)	0.823
Gastrointestinal health (n, %)	Worse: 157 (32.5)	Worse: 59 (36.6)	0.412
	Better: 327 (67.5)	Better: 101 (62.7)	
SDOH variables			
Race/Ethnicity			0.413
Non-Hispanic White	356 (73.3)	121(75.0)	
Non-Hispanic Black	53 (10.9)	18 (11.0)	
Non-Hispanic Other	6 (1.2)	2 (1.2)	
Hispanic	69 (14.2)	20 (12.8)	
Marital status			0.541
Married/Partnered	329 (68.1)	110 (68.3)	
Divorced/Widowed/Single	155 (31.9)	51 (31.7)	
Education			0.112
High school or less	247 (51.0)	80 (49.7)	
College or technical school	130 (26.9)	44 (27.3)	
Graduate school	107 (22.1)	37 (23.0)	
Household income (yr.)			0.353
Less than USD 25,000	169 (34.9)	57 (35.4)	
USD 25,000 to <USD 55,000	150 (31.0)	51 (31.7)	
USD 55,000 to <USD 75,000	45 (9.2)	17 (10.6)	
USD 75,000 and over	107 (22.1)	33 (20.5)	
Poverty–Income ratio (PIR) < 1 (yes): annual household income below the poverty level.	193 (39.7)	60 (37.3)	0.423
Food insecurity (yes)	42 (8.6)	13 (8.1)	0.879
Cancer health behaviors (yes)			
Current smoking status	86 (17.7)	31 (19.3)	0.114
Current heavy alcohol use	86 (17.7)	21 (13.0)	0.198
Regular physical activity	286 (58.8)	76 (47.2)	0.108
Diet quality (HEI-2015 score, 0–100, mean ± SE)	48.8 (12.3)	48.9 (8.3)	0.103

Note: Characteristics of variables were described using weighted means with standard error (SE) for continuous variables and unweighted N with weighted % for categorical variables, based on weighted NHANES 1999–2002 participants. ^a^ Training dataset samples n: weighted population n = 13,186,077. ^b^ Test dataset samples n: weighted population n = 4,386,277. *p*-values < 0.05 is considered the statistical significance level, based on either using the two-sample independent *t*-test or chi-square test.

**Table 2:** Factors related to GI health conditions in the training dataset.

Total Cancer Survivors (N = 484) n (%) Otherwise Specified	GI Health (n, %)		
	Better (n = 327, 67.5%)	Worse (n = 157, 32.5%)	*p*
Age (years), mean ± SE, range	63.3 (10.9)	66.4 (11.2)	47.4, **0.031**
Female (n,%)	153 (47)	103(65)	6.1, **0.013**
Modified comorbidity index (≥2) (n,%)	133(41)	71 (45)	5.4, **0.043**
Types of cancers (n,%)	Skin: 65 (20.1)	Skin: 31(19.8)	12.1, 0.100
	GU: 62 (19)	GU: 26 (16.2)	
	Breast: 53 (16.3)	Breast: 27(17.3)	
	Ovary–Uterine: 37 (11.3)	Ovary–Uterine: 18 (11.5)	
	Head and Neck: 31 (9.5)	Head and Neck: 17 (10.9)	
	GI: 27 (8.5)	GI: 15 (9.3)	
	Lung: 13 (4.1)	Lung: 8 (5.2)	
	Hematological: 36 (11.2)	Hematological: 15 (9.8)	
Body mass index (BMI), kg/m^2^, mean ± SE	31.1 (0.1)	30.1 (0.4)	0.982
BMI, kg/m^2^ (n, %) < 25	102 (31.3)	47 (29.8)	0.853
25 ≤ BMI < 30	96 (29.5)	48 (30.3)	
30 > BMI	128 (39.8)	62 (39.9)	
WBC (k/μL), normal (4–11 k/μL), mean ± SE	5.4 (1.1)	8.5 (1.5)	146.3, **0.046**
CRP (mg/dL), normal (<0.3 mg/dL), mean ± SE	0.4 (0.8)	1.0 (1.1)	238.4, **0.001**
Telomere lengths (kb), mean ± SE	0.97 (0.2)	0.64 (0.3)	85.1, **0.013**
SDOH variables			
Race/Ethnicity			24.2, **0.039**
Non-Hispanic White	260 (80.3)	122 (77.3)	
Non-Hispanic Black	35 (10.7)	17 (10.5)	
Non-Hispanic Other	5 (1.5)	2 (1.5)	
Hispanic	24 (7.5)	17 (10.7)	
Marital status			3.6, 0.730
Married/Partnered	220 (67.9)	104 (65.6)	
Divorced/Widowed/Single	104 (32.1)	54 (34.4)	
Education			16.6, 0.502
High school or less	158 (48.8)	81 (51.1)	
College or technical school	88 (27.1)	41 (25.8)	
Graduate school	78 (24.1)	36 (23.1)	
Household income (yr.)			8.43, **0.038**
Less than USD 25,000	114 (35.3)	58 (36.8)	
USD 25,000 to <USD 55,000	100 (31.0)	45 (28.3)	
USD 55,000 to <USD 75,000	50 (15.4)	26 (16.4)	
USD 75,000 and over	59 (18.3)	29 (18.5)	
Poverty–Income ratio (PIR) < 1 indicating a high poverty level (yes): annual household income below the poverty level.	113 (34.9)	59 (37.6)	18.01, **<0.001**
Food insecurity (yes)	18 (5.6)	13 (8.0)	17.01, **0.021**
Cancer health behaviors (yes)			
Current smoking status	53 (16.3)	31 (19.5)	13.1, 0.080
Current heavy alcohol use	49 (15.2)	34 (21.3)	37.01, **<0.001**
Regular physical activity	189 (58.3)	61 (38.5)	52.4, **0.035**
Diet quality (HEI-2015 score, 0–100, mean ± SE)	52.5 (5.6)	47.3 (7.5)	**56.1, 0.038**

Note: Characteristics of variables were described using weighted means with standard error (SE) for continuous variables and unweighted N with weighted % for categorical variables, based on weighted NHANES 1999–2002 participants. *p*-values are in bold if they are <0.05, as this is considered the statistical significance level, based on either using the two-sample independent *t*-test or chi-square test.

**Table 3:** Model performance evaluation indices between the training and test datasets with five-fold cross-validation.

Model	AUC	Accuracy	Precision	Sensitivity (Recall)	Specificity	F1 Score
Training Dataset (mean, 95% CI)						
LR	0.7918 (0.69–0.83)	0.7192 (0.61–0.74)	0.7214 (0.67–0.74)	0.8978 (0.87–0.90)	0.4197 (0.39–0.53)	0.8111 (0.78–0.83)
SVM	0.7994 (0.76–0.82)	0.7112 (0.68–0.75)	0.7753 (0.75–0.78)	0.7585 (0.73–0.77)	0.6321 (0.61–0.65)	0.7668 (0.71–0.79)
Decision Tree	0.9738 (0.66–0.97)	0.9089 (0.66–0.93)	**0.9340 ** (0.71–0.97)	0.9195 (0.76–0.92)	**0.8912 ** (0.79–0.95)	0.9267 (0.74–0.97)
RF	**0.9842 ** (0.78–0.99)	**0.9341** (0.74–0.98)	0.9213 (0.77–0.95)	**0.9783** (0.84–0.99)	0.8601 (0.57–0.89)	**0.9489 ** (0.80–0.98)
GBM	0.8952 (0.81–0.93)	0.7907 (0.75–0.86)	0.7867 (0.76–0.89)	0.9133 (0.87–0.97)	0.5855 (0.54–0.65)	0.8453 (0.81–0.87)
XGBoost	0.8929 (0.75–0.92)	0.7755 (0.73–0.87)	0.9195 (0.75–0.97)	0.5544 (0.52–0.84)	0.5544 (0.52–0.65)	0.8414 (0.80–0.88)
Test Dataset (mean, 95% CI)						
LR	0.7904 (0.69–0.83)	0.7287 (0.68–0.75)	0.7447 (0.72–0.77)	0.8642 (0.84–0.90)	0.5312 (0.42–0.56)	0.8012 (0.71–0.86)
SVM	0.7774 (0.76–0.80)	0.7054 (0.62–0.72)	0.7609 (0.72–0.79)	0.7407 (0.71–0.86)	0.6458 (0.61–0.68)	0.7595 (0.74–0.79)
Decision Tree	0.6480 (0.61–0.77)	0.6512 (0.63–0.70)	0.7093 (0.65–0.83)	0.7531 (0.71–0.79)	0.4792 (0.44–0.53)	0.7305 (0.64–0.82)
RF	0.7760 (0.68–0.86)	0.7364 (0.64–0.83)	0.7640 (0.71–0.82)	0.8395 (0.74–0.92)	0.7425 (0.67–0.82)	0.8000 (0.72–0.88)
GBM	**0.8035 ** (0.71–0.89)	**0.7442** (0.71–0.79)	**0.7792** (0.75–0.82)	0.8642 (0.82–0.91)	**0.7626 ** (0.68–0.81)	**0.8092 ** (0.68–0.92)
XGBoost	0.7834 (0.75–0.81)	0.7287 (0.62–0.77)	0.7500 (0.71–0.79)	0.8519 (0.76–0.94)	0.5208 (0.42–0.55)	0.7977 (0.76–0.84)

Note: AUC: area under the receiver operating characteristics (ROC) curve, known as the AUC; GBM: gradient boosting machine; LR: logistic regression; RF: random forest; SVM: support vector machine; XGBoost: extreme gradient boosting. Scores for each model performance evaluation index are averaged across folds. Bold fonts within the table indicate the highest score for the respective index.

## Data Availability

The data utilized in this study were obtained from the National Health and Nutrition Examination Survey (NHANES). NHANES data are publicly available and can be accessed through the Centers for Disease Control and Prevention (CDC) website at NHANES data. The datasets include comprehensive health and nutritional information collected from a representative sample of the US population. Researchers and the public can freely download and use these data for analysis and research purposes.

## Supplementary Materials

The following supporting information can be downloaded at: https://www.mdpi.com/article/10.3390/ijerph21121694/s1: The list of the tuned hyperparameters, copyright permission of the conceptual framework, data distribution, and McNemar’s test for model performance comparison.
