# Supplementary material for: A Machine Learning Classification Model for Gastrointestinal Health in Cancer Survivors: Roles of Telomere Length and Social Determinants of Health
Source: Int J Environ Res Public Health. 2024 Dec 19;21(12):1694. doi: 10.3390/ijerph21121694 (PMC11675289; doi:10.3390/ijerph21121694)
Supplement: Supplementary file 1 [file ijerph-3292058-supplementary.pdf › ijerph-3292058-supplementary.pdf]

Supplementary Materials

1. List of The Tuned Hyperparameters

Logistic Regression (LR)

- **C**: Uniform distribution between 0.01 and 10
- **Penalty**: ['l1', 'l2', 'elasticnet', 'none']
- **Solver**: ['newton-cg', 'lbfgs', 'liblinear', 'sag', 'saga']

Support Vector Machine (SVM)

- **C**: Uniform distribution between 0.01 and 10
- **Kernel**: ['linear', 'poly', 'rbf', 'sigmoid']
- **Degree**: Random integer between 2 and 5 (for polynomial kernel)
- **Gamma**: ['scale', 'auto']

Decision Tree

- **Max Depth**: Random integer between 1 and 20
- **Min Samples Split**: Random integer between 2 and 20
- **Min Samples Leaf**: Random integer between 1 and 20
- **Criterion**: ['gini', 'entropy']

Random Forest (RF)

- **N Estimators**: Random integer between 100 and 500
- **Max Depth**: Random integer between 1 and 20
- **Min Samples Split**: Random integer between 2 and 20
- **Min Samples Leaf**: Random integer between 1 and 20
- **Bootstrap**: [True, False]

Gradient Boosting Machine (GBM)

- **N Estimators**: Random integer between 100 and 500
- **Learning Rate**: Uniform distribution between 0.01 and 0.3
- **Max Depth**: Random integer between 1 and 20
- **Min Samples Split**: Random integer between 2 and 20
- **Min Samples Leaf**: Random integer between 1 and 20

XGBoost

- **N Estimators**: Random integer between 100 and 500
- **Learning Rate**: Uniform distribution between 0.01 and 0.3
- **Max Depth**: Random integer between 1 and 20
- **Subsample**: Uniform distribution between 0.5 and 1.0
- **Colsample Bytree**: Uniform distribution between 0.5 and 1.0

2. Copyright Permission

9/26/2024.
Dear Professor Dr. Robert Gobbens

We understand that you are the copyright holder for the **Integral Conceptual Model of Frailty.** We want to use and adapt your model applicable to our manuscript, and we have previously received your email confirmation.

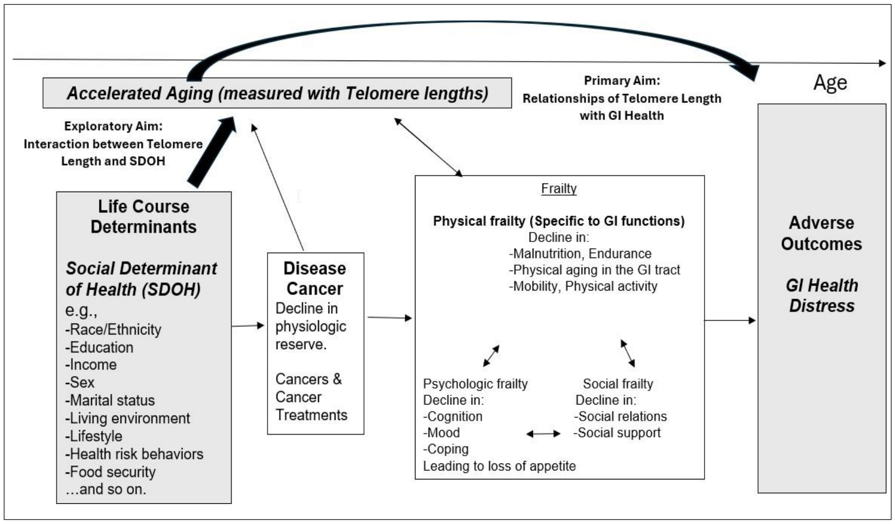

To have your official permission, please sign this permission letter and return your signed copy to us by email.
We appreciate your consideration of our permissions request.
Sincerely,

Claire (Jungyoun), Han, Ph.D., DNP, ARNP
Pronouns:she/her/hers
Assistant Professor, College of Nursing
Center for Healthy Aging, Self-Management and Complex Care
The Ohio State University
377, Newton Hall, 1585 Neil Avenue, Columbus, OH 43210

Member, Cancer Prevention and Control Program
The Ohio State University Comprehensive Cancer Center
James Cancer Hospital and Solove Research Institute, Columbus, OH 43210
han.1985@osu.edu

Professor Dr. Robbert Gobbens
Full Professor
TSB: Tilburg School of Social and Behavioral Sciences
TSB: Tranzo
R.J.J.Gobbens@tilburguniversity.edu

By signing below, I warrant that I have the right to grant the permission requested in this letter, and that I provide you with that permission.

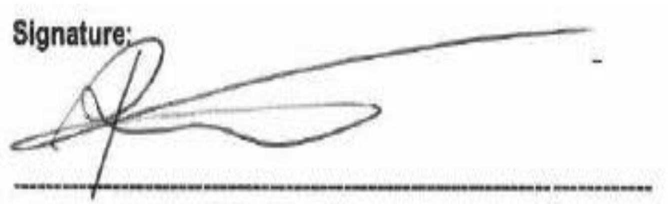

Date: 9/26/2024

3. Data Distributions

We present the normal distributions of continuous variables among relevant input features of GI health.

Training set (n = 75% of total sample, n = 484)	Test set (n = 25% of total sample, n = 161)

Age (years old)

Training set

Tests of Normality

	Kolmogorov-Smirnov[a]			Shapiro-Wilk		
	Statistic	df	Sig.	Statistic	df	Sig.
age	.025	484	.200*	.998	484	.897

*. This is a lower bound of the true significance.

a. Lilliefors Significance Correction

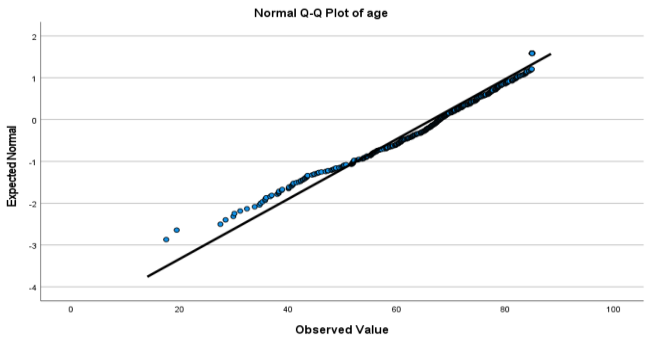

Test set

Tests of Normality

	Kolmogorov-Smirnov[a]			Shapiro-Wilk		
	Statistic	df	Sig.	Statistic	df	Sig.
age	.045	161	.232*	.999	161	.897

*. This is a lower bound of the true significance.

a. Lilliefors Significance Correction

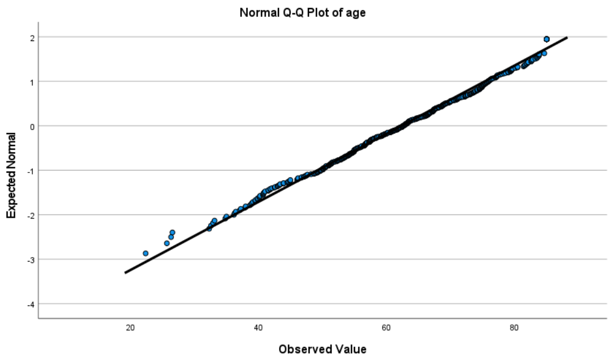

WBC (k/ul)

Training set

Tests of Normality

	Kolmogorov-Smirnov[a]			Shapiro-Wilk		
	Statistic	df	Sig.	Statistic	df	Sig.
age	.032	484	.200*	.997	484	.686

*. This is a lower bound of the true significance.

a. Lilliefors Significance Correction

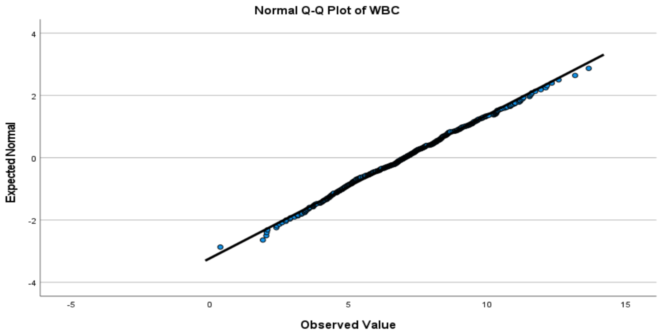

Test set

Tests of Normality

	Kolmogorov-Smirnov[a]			Shapiro-Wilk		
	Statistic	df	Sig.	Statistic	df	Sig.
age	.034	161	.211*	.997	161	.543

*. This is a lower bound of the true significance.

a. Lilliefors Significance Correction

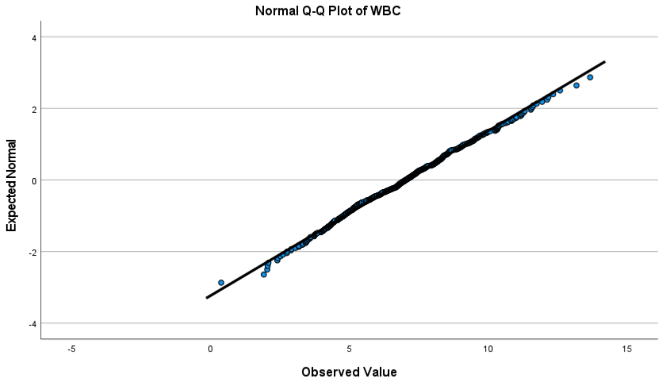

CRP (mg/dl)

Tests of Normality

	Kolmogorov-Smirnov[a]			Shapiro-Wilk		
	Statistic	df	Sig.	Statistic	df	Sig.
age	.040	484	.064*	.997	484	.159

*. This is a lower bound of the true significance.

a. Lilliefors Significance Correction

Tests of Normality

	Kolmogorov-Smirnov[a]			Shapiro-Wilk		
	Statistic	df	Sig.	Statistic	df	Sig.
age	.022	161	.192*	.999	161	.491

*. This is a lower bound of the true significance.

a. Lilliefors Significance Correction

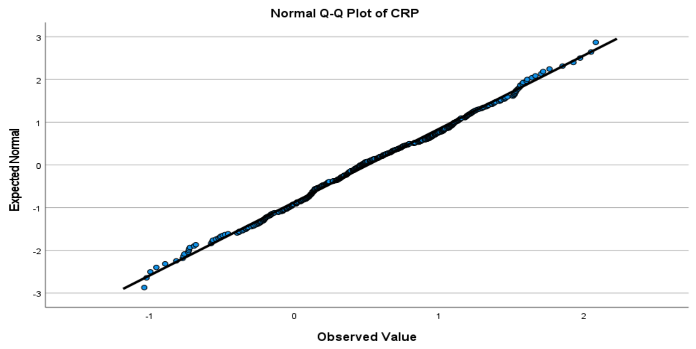

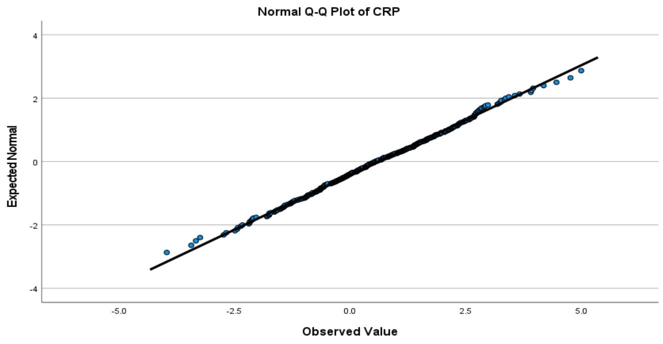

Telomere Lengths (kb)

Tests of Normality

	Kolmogorov-Smirnov[a]			Shapiro-Wilk		
	Statistic	df	Sig.	Statistic	df	Sig.
age	.036	484	.164*	.996	484	.366

*. This is a lower bound of the true significance.

a. Lilliefors Significance Correction

Tests of Normality

	Kolmogorov-Smirnov[a]			Shapiro-Wilk		
	Statistic	df	Sig.	Statistic	df	Sig.
age	.034	161	.210*	.991	161	.122

*. This is a lower bound of the true significance.

a. Lilliefors Significance Correction

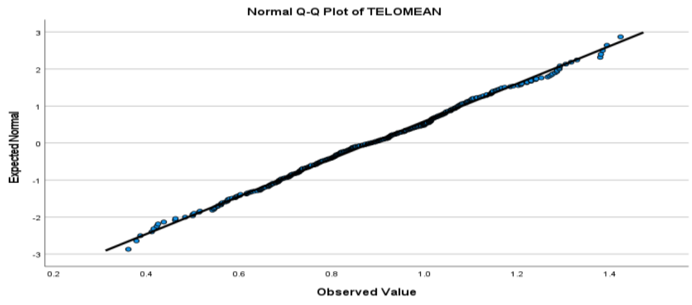

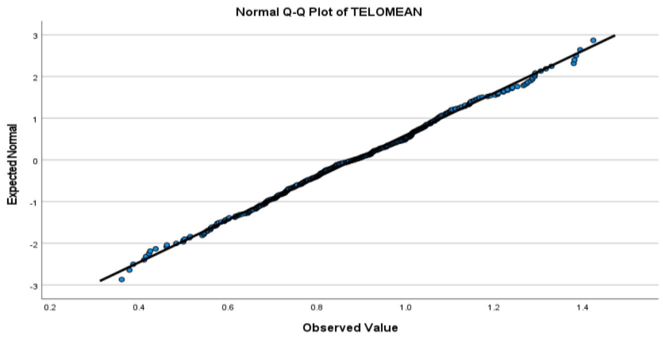

Diet quality (HEI)

Tests of Normality

	Kolmogorov-Smirnov[a]			Shapiro-Wilk		
	Statistic	df	Sig.	Statistic	df	Sig.
age	.023	484	.200*	.997	484	.601

*. This is a lower bound of the true significance.

a. Lilliefors Significance Correction

Tests of Normality

	Kolmogorov-Smirnov[a]			Shapiro-Wilk		
	Statistic	df	Sig.	Statistic	df	Sig.
age	.036	161	.200*	.992	161	.525

*. This is a lower bound of the true significance.

a. Lilliefors Significance Correction

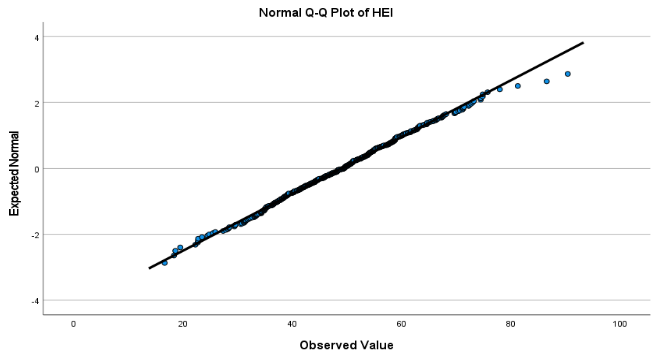

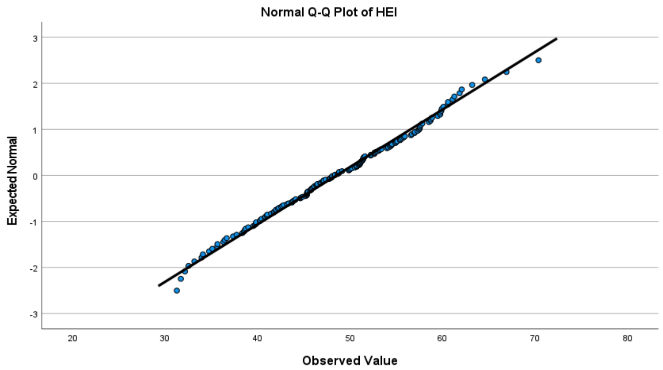

4. Models’ performance comparison by using McNemar’s statistical test.

Model	AUC	*p*	Accuracy	*p*	Precision	*p*	Sensitivity (Recall)	*p*	Specificity	*p*	F1 Score	*p*
**Training Dataset (mean)**												
**Decision Tree**	0.9738	**0.045**	0.9089	**0.041**	0.9340	**0.048**	0.9195	**0.002**	0.8912	**0.013**	0.9267	**0.012**
**RF**	0.9842		0.9341		0.9213		0.9783		0.8601		0.9489	
**Test Dataset (mean)**												
**LR**	0.7904	**0.041**	-	-	-	-	0.8642	Not applicable	-	-	0.8012	0.053
**RF**	-		0.7364	0.051	0.7640	**0.042**	-		0.7425	**0.042**	-	
**GBM**	0.8035		0.7442		0.7792		0.8642		0.7626		0.8092	

Note. AUC: the area under the receiver operating characteristics (ROC) curve known as the AUC; GBM: Gradient boosting machine; LR: Logistic regression, RF: Random forest. Since our study focuses on identifying the optimal AI model selections, we only compared the top two models with the highest values in each performance index. Bold fonts within the table indicate the statistical significance of each model's performance index between the models by using McNemar’s statistical test ($p < .05$).
